# Odontoblast-Like Cells Differentiated from Dental Pulp Stem Cells Retain Their Phenotype after Subcultivation

**DOI:** 10.1155/2018/6853189

**Published:** 2018-02-19

**Authors:** Paula A. Baldión, Myriam L. Velandia-Romero, Jaime E. Castellanos

**Affiliations:** ^1^Grupo de Investigaciones Básicas y Aplicadas en Odontología, Universidad Nacional de Colombia, Bogotá, Colombia; ^2^Grupo de Virología, Universidad El Bosque, Bogotá, Colombia

## Abstract

Odontoblasts, the main cell type in teeth pulp tissue, are not cultivable and they are responsible for the first line of response after dental restauration. Studies on dental materials cytotoxicity and odontoblast cells physiology require large quantity of homogenous cells retaining most of the phenotype characteristics. Odontoblast-like cells (OLC) were differentiated from human dental pulp stem cells using differentiation medium (containing TGF-*β*1), and OLC expanded after trypsinization (EXP-21) were evaluated and compared. Despite a slower cell growth curve, EXP-21 cells express similarly the odontoblast markers dentinal sialophosphoprotein and dentin matrix protein-1 concomitantly with RUNX2 transcripts and low alkaline phosphatase activity as expected. Both OLC and EXP-21 cells showed similar mineral deposition activity evidenced by alizarin red and von Kossa staining. These results pointed out minor changes in phenotype of subcultured EXP-21 regarding the primarily differentiated OLC, making the subcultivation of these cells a useful strategy to obtain odontoblasts for biocompatibility or cell physiology studies in dentistry.

## 1. Introduction

Odontoblasts are highly specialized cells that produce both collagen and noncollagen proteins to build the dentinal extracellular matrix. They are also the pulp first responders against exogenous stimulus or dental materials [1, 2]. Due to their postmitotic phenotype, odontoblasts are difficult to culture [3, 4]; thus, biochemical or toxicologic studies assessing their use in dental materials have been limited. To overcome such difficulties, studies on materials toxicity have been developed on primary gingival fibroblasts [5], primary human or mouse undifferentiated mesenchymal stem cells, and immortalized cell lines [6, 7].

However, the genotype, phenotype, or cell responses in these* in vitro* models could differ from actual odontoblast cell responses due to genetic changes [8] or environmental adaptations of the cell lines, which complicates the interpretation of cytotoxicity results.

Human dental pulp stem cells (hDPSC) have been isolated as adherent mononucleated cells with* in vitro* differentiation capacity toward several lineages, including odontoblasts, osteoblasts, adipocytes, and neural cells. Differentiated odontoblasts establish a structure known as the dentin-pulp complex; therefore, these cells have been proposed as tools in dental regeneration and repair [9].

Obtaining homogeneous odontoblast-like cells (OLC) differentiated from hDPSC that retain their phenotype after monolayer detachment, reseeding and expansion could benefit (I) studies on gene expression and the specific protein synthesis involved in the secretion and mineralization of the extracellular matrix (ECM) and (II) studies on the cellular response after challenge with dental biomaterials. Expanded cells enable a sufficient number of cells to make replicates for toxicity and cell metabolism studies, overcoming the heterogeneity and lack of reproducibility that frequently appear during the use of primarily differentiated odontoblasts.

Differentiated cultured cells are well known to revert to an undifferentiated phenotype after subcultivation [10], making it difficult to perform studies that require large numbers of cells. Therefore, data on culture conditions, proliferation rates, and the differentiation status of subcultured cells are important for comparing and validating experiments using primary differentiated cultures. This study aimed to standardize an* in vitro* cell model for differentiating hDPSC into OLC, as well as describe and compare the phenotype after the detachment and reexpansion of differentiated cells.

The results showed that hDPSC differentiate to OLC in the presence of mineralizing medium enriched with TGF-*β*1. The detachment and subcultivation of differentiated cells cause minor changes in protein and gene expression in relation to primary differentiated OLC; therefore, subculture in the differentiation medium allows the cells to retain the OLC phenotype. The reexpanded OLC may be a useful tool for studying the cell physiology of dentin-pulp complex formation and for use in biocompatibility studies of dental biomaterials.

## 2. Materials and Methods

### 2.1. Primary Culture of Human Dental Pulp

The study protocol was revised and approved by the Ethics Committee of Facultad de Odontología of Universidad Nacional de Colombia (CIE-233-14). Teeth collection, handling, and disposal were carried out in accordance with ethical standards of national and international legislation, and all volunteers signed the informed consent form before surgery. The third molars of young individuals (14–18 years old) were surgically extracted due to orthodontic considerations and processed following the protocol suggested by Gronthos et al. [11]. Teeth were decontaminated with 0.5% sodium hypochlorite and sectioned to obtain the pulp, which was cut into small fragments and digested overnight with collagenase (3 mg/mL) (Sigma-Aldrich; St Louis, MO, USA) and dispase (4 mg/mL) (Gibco; Thermo Fisher Scientific; Bremen, Germany) prepared in low-glucose DMEM supplemented with 10% fetal bovine serum (FBS) (Hyclone; Thermo Fisher Scientific; Bremen, Germany), penicillin (100 U/mL), and streptomycin (100 *μ*g/mL) at 37°C in a 5% CO_2_ incubator. The cell suspension was centrifuged, and the pellet was resuspended in low-glucose DMEM supplemented with 10% FBS and seeded in 25 cm^2^ culture flasks until they reached 80% confluence. Cells were detached with trypsin plus EDTA and reseeded in 75 cm^2^ culture flasks. At fourth passage (split), cells from independent donors were evaluated by flow cytometry to detect mesenchymal cell markers. Independent cultures from three donors and three replicas were analyzed (*n* = 9).

### 2.2. Flow Cytometry

The criteria of the International Society for Cell Therapy for mesenchymal stem cells [12] were used to determine the phenotype of hDPSC. A staining cocktail for phenotyping was used on 1 × 10^6^ cells (Miltenyi Biotec; Bergisch Gladbach, Germany). Markers included antibodies targeting CD73, CD90, CD105, CD14, CD20, CD34, and CD45. Cells lacking primary antibodies were incubated with mouse isotype controls (FITC-IgG_1_, PE-IgG_1_, APC-IgG_1_, PerCP-IgG_1_, and PerCP-IgG_2a_). Fluorescence of stained cells was captured in a FACSCalibur cytometer (BD Biosciences; San Jose, CA, USA), and after the acquisition of 100.000 events, data were analyzed using FCS Express software. Data are expressed as the percentage of cells positive for each marker.

### 2.3. Odontoblast-Like Cell Differentiation

Cell differentiation was performed using the protocol reported by Teti et al. (2013) with minor modifications [13]. Briefly, pulp mesenchymal stem cells were treated with odontogenic induction medium, DMEM supplemented with 10% FBS, antibiotics, 0.1 *μ*M dexamethasone (Sigma-Aldrich), 5 mM *β*-glycerophosphate (Santa Cruz, CA, USA), 50 *μ*g/mL ascorbic acid (Sigma-Aldrich), and 10 ng/mL TGF-*β*1 (Sigma-Aldrich) for 7, 14, or 21 days at 37°C in a 5% CO_2_ incubator. After 21 days of treatment, differentiated odontoblast-like cells (OLC) were detached using trypsin/EDTA (0.25%/0.5 mM) and reseeded in new flasks using the same odontogenic induction media; these cells were named “expanded at 21 days” (EXP-21). To assess whether cryopreserved EXP-21 cells retain their differentiated phenotype, the cells were resuspended in 10% FBS supplemented DMEM containing 10% dimethyl sulfoxide at a concentration of 1-2 × 10^6^ cells/mL and then frozen at −80°C overnight and transferred to liquid nitrogen for undefined storage. The cells were thawed in a 37°C water bath in differentiation medium and cultured again for 24 h with differentiation medium. Both the OLC and EXP-21 cells (fresh and thawed) were characterized based on their phenotype as described below. Odontoblast markers were evaluated by immunohistochemistry at 7, 14, and 21 days after reseeding in fresh and thawed EXP-21 cells. For extracellular matrix formation and population doubling time, cells were maintained for 14 and 21 additional days, respectively, in the same manner as for OLC. Human dental pulp mesenchymal cells maintained with basal medium were considered negative controls.

### 2.4. Evaluation of Odontoblast Gene Expression by Quantitative RT-PCR

TRIzol (Ambion; Life Technologies, Carlsbad, California) was used to isolate total RNA from OLC at 7, 14, and 21 days after differentiation and from EXP-21 cells. With 200 ng of RNA per sample, RT-PCR was performed using the SYBR Green One-Step Real-Time RT-PCR Master Mix system (Invitrogen; Grand Island, NY, USA) in the CFX96 Real-Time Thermal Cycler detection system (Bio-Rad; Hercules, CA, USA). Amplification conditions were as follows: 15 min retrotranscription at 50°C, 4 min at 94°C and 40 amplification cycles of 20 s at 94°C, 20 s at 60–62°C, and 20 s at 72°C. Dentinogenic mRNA markers were quantitated using a specific primer set (Table 1); the genes evaluated were osteopontin (OPN), osterix (OSX), alkaline phosphatase (ALP), type I-collagen (COL-I), runt-related transcription factor 2 (RUNX2), dentin sialophosphoprotein (DSPP), dentin matrix protein-1 (DMP-1), and *β*-Actin (*β*-ACT). DSPP and DMP-1 as specific odontogenic markers were further evaluated in thawed EXP-21 cells to demonstrate phenotype maintenance after cryopreservation. PCR efficiencies were calculated using LinRegPCR (Academic Medical Center, AMC, Amsterdam, Netherlands), and relative gene quantitation was performed following Schefe's method [14].

### 2.5. Calculation of Cell Growth Curves and Population Doubling Time (PDT)

The three cell types (hDPSC, OLC, and EXP-21 cells) were seeded in triplicate in 48-well microplates (8.000 cells/well) in the appropriate culture medium and were trypsinized and counted in a hemocytometer with trypan blue daily for 21 days. Data regarding the cell number and the number of days in culture were plotted, and PDT was estimated using the formula (*t*2 − *t*1)/3.32 × (log⁡*n*2 − log⁡*n*1), where *t* is the number of days in culture and *n* is the cell number; data output was confirmed using Doubling Time software [15].

### 2.6. Immunocytochemical Detection of Odontogenic Markers

The immunocytochemistry protocol has been reported previously [16]. Cells were seeded on poly-L-lysine-treated glass coverslips (8.000 cells/well). After reaching 30% confluence, the cells were differentiated for 21 days as described above. Monolayers were fixed with paraformaldehyde (4%), permeabilized, and incubated with one of two primary antibodies: first, polyclonal anti-DSPP (Abcam, Cambridge, MA, USA), which is specific to the DSPP N-terminus corresponding to the natural cleavage fragment dentin sialoprotein (DSP); second, anti-DMP-1 (Sigma-Aldrich). Both were prepared in blocking buffer at 1 : 50 dilution. Goat anti-rabbit IgG biotinylated antibody was added at room temperature, the samples were washed, and peroxidase-coupled streptavidin (Thermo Fisher Scientific) was added. Specific binding was visualized using H_2_O_2_ and 3,3′-diaminobenzidine tetrahydrochloride chromogen. Nuclei were counterstained with hematoxylin, and the cells were photographed using a Zeiss Axio Imager A2 microscope (Gottingen, Germany). Other culture sets were processed for immunofluorescence using Alexa Fluor 594 coupled streptavidin (Thermo Fisher Scientific). The nuclei were counterstained with Hoechst, observed in an Axio Imager A2 microscope (Zeiss, Germany) and analyzed with the AxioVision software.

### 2.7. ECM Protein Adhesion Assay

The CytoSelect system (Cell Biolabs, Inc.; San Diego, CA, USA), which uses a 48-well microplate coated with five different ECM proteins, was used to test the cell substrate adhesion of the EXP-21 cultures. Trypsinized cells (7.000 cells/well) were seeded in serum-free medium in each of the coated wells at 37°C for 1 h, followed by washing with PBS and staining with Coomassie blue for 10 min. Cells were destained with acetic acid, and absorbance was measured at 560 nm in a microplate reader (Tecan, Infinite M200; Männedorf, Switzerland).

### 2.8. Measurement of Alkaline Phosphatase Activity

The hDPSC were cultivated in maintenance medium (low-glucose DMEM supplemented with 10% FBS and antibiotics) at 10.000 cells/well in a 24-well microplate until they reached 60% confluence. The EXP-21 cells were maintained for 24 h until adherence in differentiation media, and OLC were differentiated over 3, 7, 14, and 21 days. During each assay period, the cells were detached, lysed in assay buffer, and centrifuged. Supernatants were transferred to a 96-well microplate to quantify the ALP activity using an Abcam kit based on the fluorogenic substrate 4-methylumbelliferyl phosphate, resulting in an increased fluorescent signal (*Ex*/*Em* = 360 nm/440 nm) when the substrate is dephosphorylated.

### 2.9. Staining of Cultures

ECM components were stained using Masson's trichrome. Cells (hDPSC, OLC, and EXP-21 cells) were seeded on poly-L-lysine-treated glass coverslips. The OLC were differentiated for 7, 14, or 21 days, and the EXP-21 cells were maintained for 14 days before ethanol/acetone fixation. The collagen fibers were then stained with 1% phosphomolybdic/phosphotungstic acid for 5 min, followed by immersion in aniline blue stain, washing for 2 min with 1% acetic acid, mounting, and photography. To evaluate calcified nodes, the cultured cells were fixed with formaldehyde and stained with 2% alizarin red pH 4.1 for 15 min (Sigma-Aldrich) and photographed. The stain was extracted for 16 h with 5% (v/v) 2-isopropanol and 10% (v/v) acetic acid solution, and absorbance was measured using a microplate reader at 550 nm. Von Kossa staining was used to detect the calcium deposits in the formaldehyde-fixed cultures. A 5% silver nitrate solution was added to the cultures for 15 min; after washing, the cultures were exposed to UV light for 1 h, and calcium phosphate nodes were counted using a Zeiss Axiovert 40 CFL microscope.

### 2.10. Statistical Analysis

Data were collected in an Excel worksheet and exported to SPSS software, version 21.0 (SPSS, Chicago, IL, USA) for analysis. Data are presented as the mean and standard deviation. Differences with *p* values less than 0.05 were considered significant. Student's *t*-test was used in series with normal distributions, and the Mann–Whitney *U* test was used to compare data with a nonparametric distribution. All of the conditions were evaluated in three independent experiments with two, three, or six replicates.

## 3. Results

### 3.1. hDPSC Were Differentiated to Odontoblast-Like Cells and Retained Their Phenotype after Detachment

**Figure 1:**
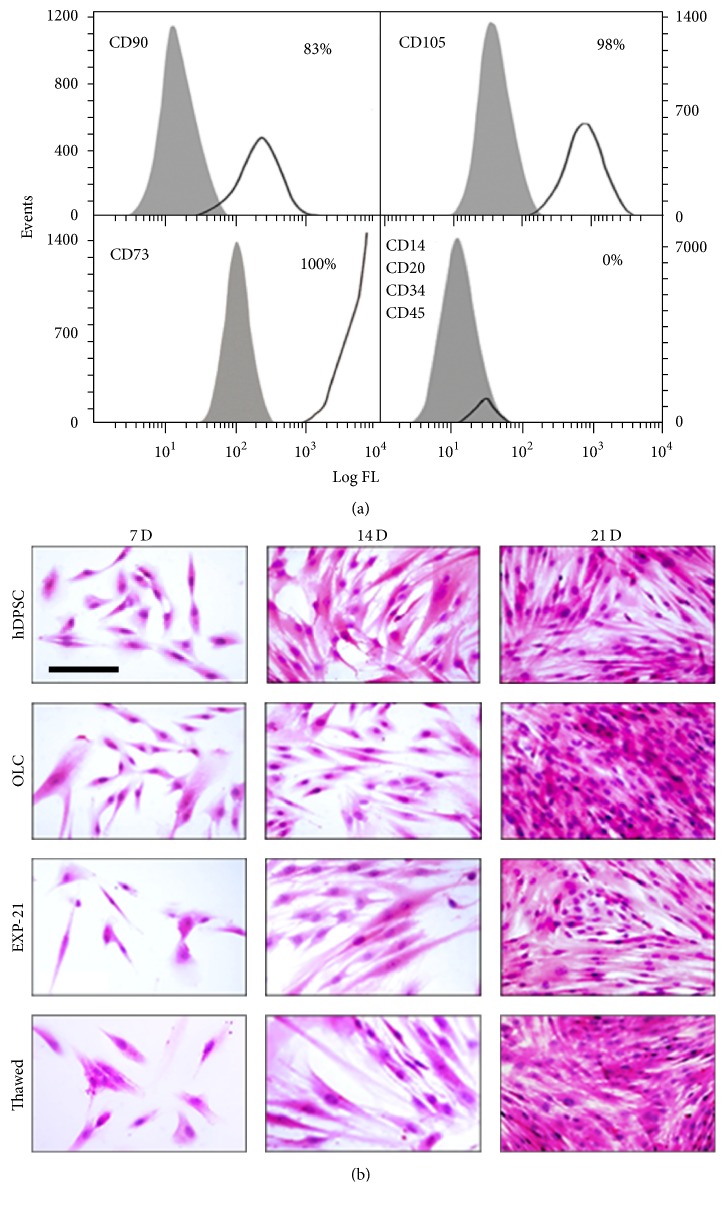
*hDPSC surface markers and morphology of the cultures*. The hDPSC were isolated from dental pulp of permanent teeth from three healthy donors. (a) The analysis by flow cytometry showed a homogeneous cell population positive for mesenchymal markers CD90, CD105, and CD73 and negative for early hematopoietic markers CD34, CD45, CD14, and CD20. Cells from fourth passage were recorded of each sample in triplicate. Data correspond to a representative experiment. (b) The hDPSC showed spindle-shape or fibroblast-like morphology with adherence to the surface and colony formation ability growing in a swirling-like pattern. During the differentiation process (OLC) and in the EXP-21, the cells maintained a typical fibroblastic-like shape with long cytoplasmic processes and were orderly arranged with a tendency to align themselves in parallel lines. The EXP-21 cells after cryopreservation, storage, and thawing showed similar morphology than their regular noncryopreserved cells. Scale bar: 200 *μ*m.

**Figure 2:**
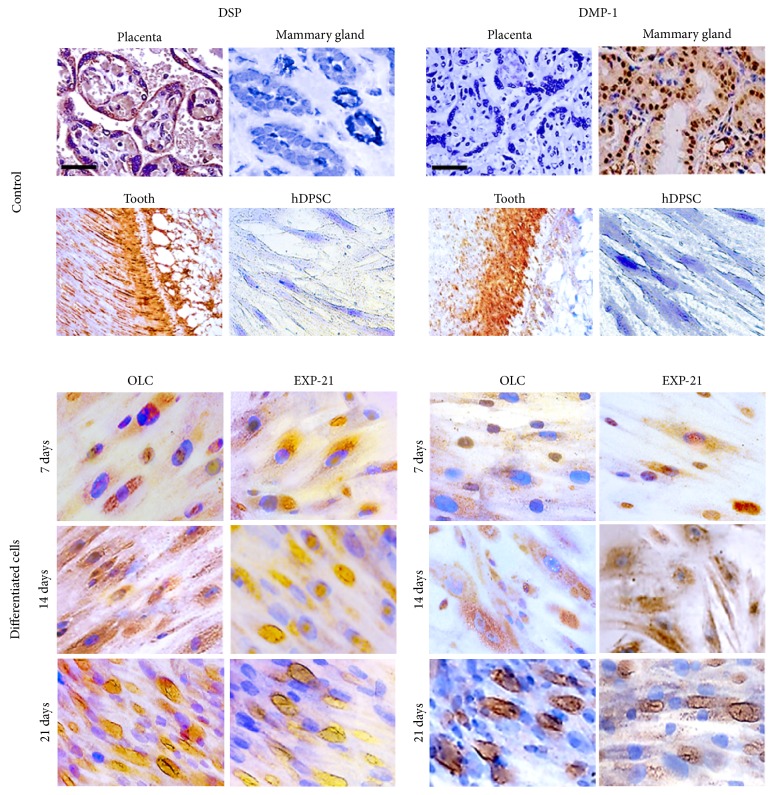
*Immunocytochemical detection of DSP and DMP-1 odontogenic markers*. Upper panel: the staining pattern in positive controls for DSP (placenta), DMP-1 (mammary gland), and dental pulp tissue positive for both markers. Mesenchymal cells are negative for these markers. Lower panel: DSP and DMP-1 staining pattern in OLC differentiating cultures for 7, 14, and 21 days and reseeded expanded cells (EXP-21) at 7, 14, and 21 days after differentiation. Note the cytoplasmic, nuclear, and late extracellular DSP staining pattern (OLC-21 and EXP-21, left panel). DMP-1 is expressed in both nuclei and cytoplasm and also in extracellular space as matrix vesicles (OLC-21 and EXP-21, right panel). Bar correspond to 100 *μ*m.

**Figure 3:**
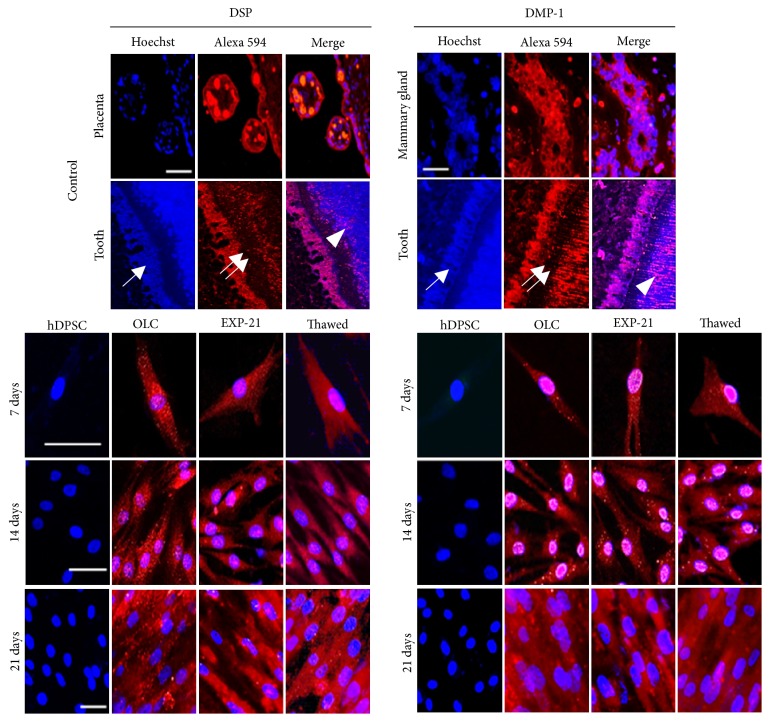
*Immunofluorescence analysis of DSP and DMP-1 localization*. Staining of respective positive controls for DSP (placenta) and DMP-1 (mammary gland). In the tooth, a layer of columnar odontoblasts (arrow) surrounds the inner surface of dentin and shows positive staining for both DSP and DMP-1; negative staining in the predentin area was observed (double arrow) and positive staining on the dentin mineralization front (arrowhead). To obtain a detailed protein marker location in cell cultures, they were processed for Alexa 594 immunofluorescence with antibodies to DSP or DMP-1 and Hoechst to stain nuclei after 7, 14, and 21 days of differentiation period (OLC) and 7, 14, and 21 days after reseeding process in fresh cells (EXP-21) and thawed EXP-21 cells (Thawed). Note the intense nuclear staining of DMP-1 and the absence of staining in mesenchymal cells. Bar corresponds to 100 *μ*m.

As expected, hDPSC showed the marker profile of mesenchymal cells (CD73+, CD90+, CD105+, CD14−, CD20−, CD34−, CD45−) (Figure 1(a)) and formed adherent clonogenic clusters after culture in flasks with maintenance medium. These fibroblast-like cells had a high proliferation rate between 7 and 21 days in culture, and differentiated cells (odontoblast-like cells, OLC) appeared with the same morphology throughout the entire culture period. The EXP-21 cells acquired a fusiform morphology and were capable of colony formation, significantly increasing the proliferation rate after reseeding for 7, 14, and 21 days of cultivation. After reaching confluence, the EXP-21 cells grew as an adherent multiple layer pattern arranged in parallel lines (Figure 1(b)). While hDPSC did not express the odontoblast phenotype markers DSPP and DMP-1, differentiating OLC highly expressed those markers beginning on day 14, and this differentiated pattern was maintained in detached and reseeded EXP-21 cells even after freeze-thaw processing (Figure 2). A low cell density per well seeding at the beginning of the differentiation stimuli enabled us to obtain a detailed protein marker location. We thus easily found in both OLC and EXP-21 cells (fresh and thawed) a cytoplasmic fluorescence and nuclear pattern for DSP, while DMP-1 was primarily located in the nuclei with less intensity in the cytoplasm. At the end of the differentiation treatment (21 days), we found both secreted DSP and DMP-1 outside the cell as part of the ECM (Figure 3).

### 3.2. Cell Growth and Proliferation Changed during Differentiation

**Figure 4:**
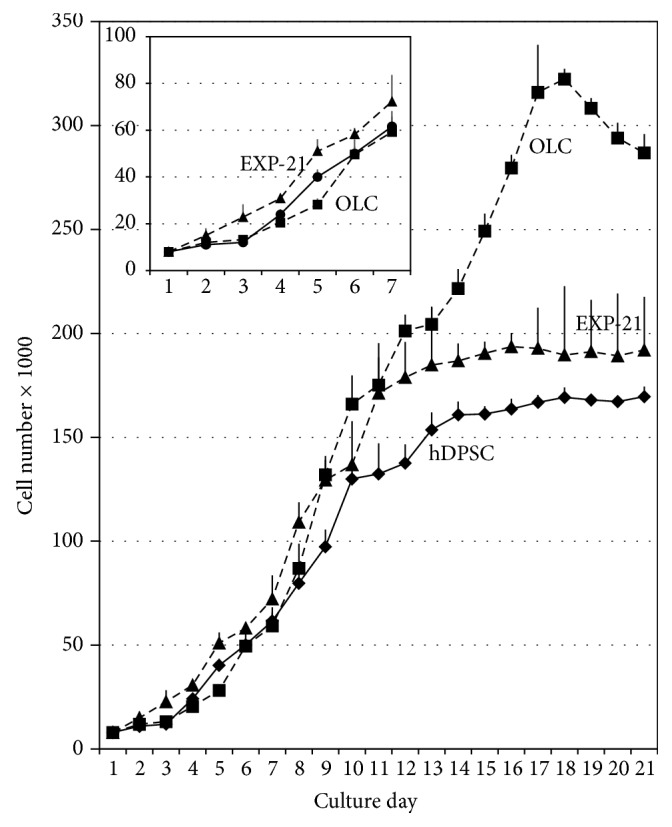
*Cell growth curves*. The hDPSC, OLC, and EXP-21 cells were detached daily for 21 days to determine the average number of cells in each cell population. hDPSC were maintained in culture medium without differentiation factors for 21 days, the OLC were stimulated with differentiation medium, and EXP-21 cells were maintained with differentiation medium for additional 21 days after detaching. Data are shown as mean ± SD and are based on three independent experiments and each was quantitated by triplicate (*n* = 9).

To evaluate the role of the differentiation medium and the detachment process, growth curves and PDT values were obtained during 21 days in the three culture types. OLC proliferation during differentiation was slow (PDT 51.6 h), causing a delay in reaching confluence and generating tightly multilayered colonies with a concomitantly higher number of cells per unit of culture area. However, the hDPSC and EXP-21 cells had similar PDT values (38.2 h and 38.8 h, resp.) when evaluated after the first 96 h of culture (Figure 4).

### 3.3. Differentiated Cells Expressed Odontogenic Genes and Markers

**Figure 5:**
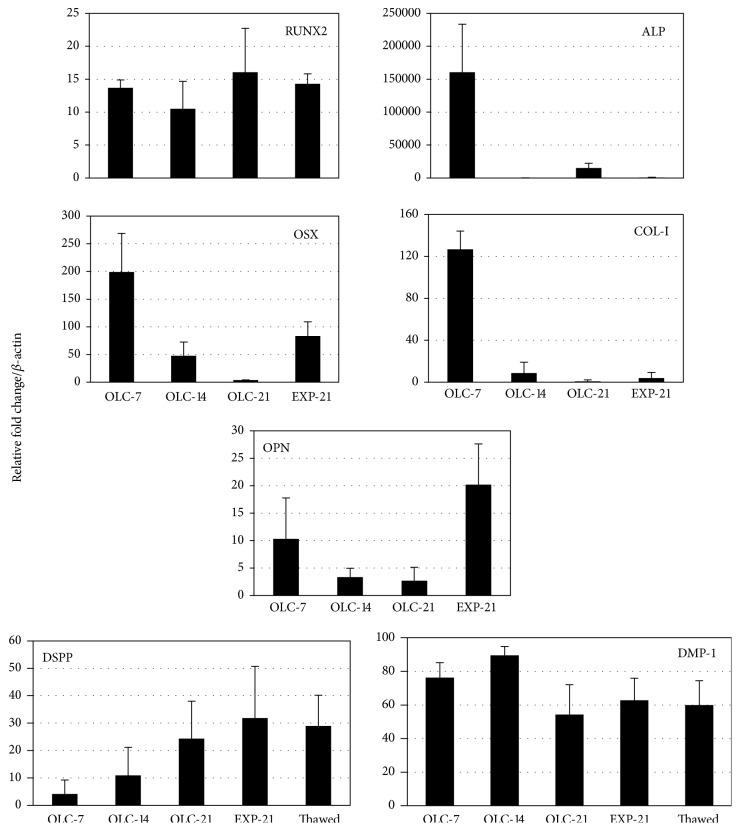
*Relative gene expression quantitation during differentiation*. Specific odontoblast and mineralizing transcripts were quantitated in differentiating OLC (7, 14, and 21 days) and in both fresh and thawed EXP-21 cells by real-time PCR considering a value of 1 in hDPSC. Data are shown as mean ± SD of three independent experiments in duplicate (*n* = 6). Runt-related transcription factor 2 (RUNX2), alkaline phosphatase (ALP), osterix (OSX), type I-collagen (COL-I), dentin sialophosphoprotein (DSPP), dentin matrix protein-1 (DMP-1), osteopontin (OPN).

RUNX2 transcription factor mRNA increased by 12–18-fold in differentiated OLC and EXP-21 cells with respect to the undifferentiated hDPSC and remained high at all of the evaluated points, whereas the OSX transcripts reached a significant peak after 7 days of OLC differentiation (200-fold change). Similarly, COL-I and ALP transcript levels were significantly higher after 7 days of differentiation (125- and 160.000-fold differences, resp.) compared with OLC at later differentiation times (14 or 21 days) or even in EXP-21 cells, which had values equivalent to those of OLC at 21 days of differentiation (OLC-21) (Figure 5).

Interestingly, the transcript level of OSX was more than threefold higher in the EXP-21 cells compared to the OLC-21 cells. Regarding the odontoblast markers, the DSPP transcripts increased by 5-fold in the OLC-7 cells and 25-fold in the OLC-21 cells compared with hDPSC. The expression peak of DMP-1 was at 14 days of differentiation, showing 90-fold more expression than the hDPSC. The EXP-21 cells maintained high DSPP (32-fold increase over hDPSC) and DMP-1 (63-fold increase over hDPSC) transcription, giving expression values that were like those in the cells after being thawed. In turn, OPN transcription was 20-fold higher in the EXP-21 cells than the OLC-21 cells (Figure 5). These data were obtained from cells of three different donors in experiments performed independently in duplicate.

### 3.4. EXP-21 Cells Use Primarily Fibronectin, Collagen, and Fibrinogen for Substrate Adherence

**Figure 6:**
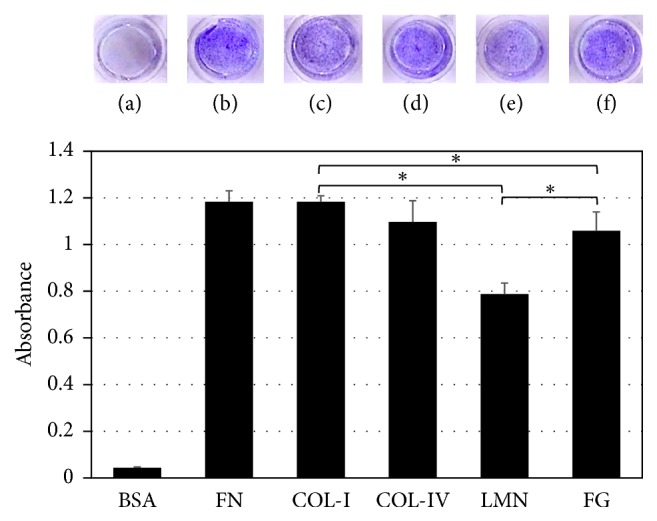
*EXP-21 cells extracellular matrix (ECM) protein adhesion assay*. Cell adhesion assay CytoSelect™ (Cell Biolabs) was employed to characterize cell attachment to ECM proteins like (b) fibronectin (FN), (c) type I-collagen (COL-I), (d) type IV-collagen (COL-IV), (e) laminin (LMN), (f) fibrinogen (FG). (a) Bovine serum albumin (BSA) is used as adhesion control. Attached cells after 1 h were stained with Coomassie blue dye which was subsequently extracted to compare the absorbances. The lowest adhesion was obtained on LMN. Data shown are mean ± SD of three independent experiments in duplicate (*n* = 6). Asterisks show significant differences (*p* < 0.05).

The CytoSelect system showed increased adherence of EXP-21 to fibronectin and to type I- and IV-collagen (25-fold higher) compared with the control substrate (BSA). A similar finding was observed in fibrinogen-coated wells but not in laminin-coated substrates. Cells obtained from different donors had a similar adherence behavior (Figure 6).

### 3.5. Differentiated OLC Mineralized the ECM

**Figure 7:**
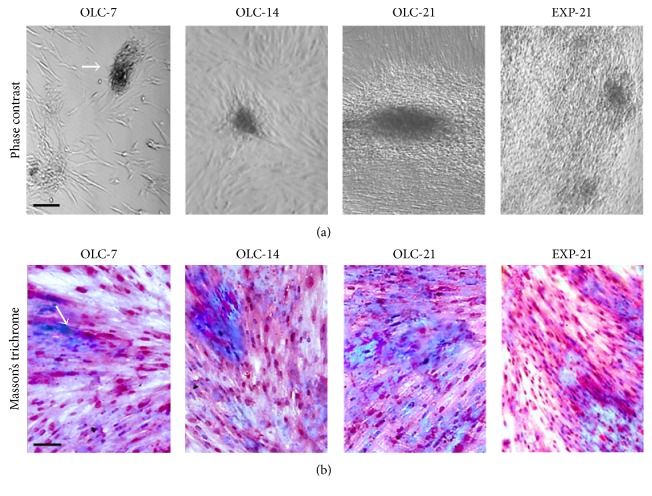
*Extracellular matrix deposition*. OLC and EXP-21 cultures were exposed to differentiation-inductive conditions. Cell aggregates (black arrow) became the centers where there was an extensive deposition of extracellular matrix components. Masson's trichrome staining (black arrow, (b)) shows the deposition of COL-I. Scale bar: 100 *μ*m.

**Figure 8:**
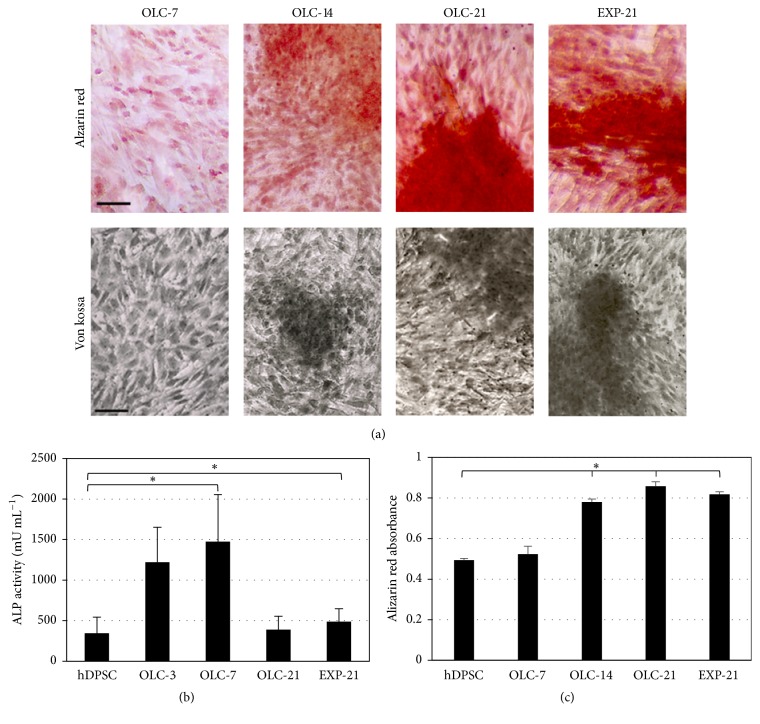
*Extracellular matrix mineralization*. (a) Mineralization was determined by alizarin red (upper panel) and von Kossa staining (lower panel). The strong staining of matrix indicates the apparent formation of calcification nodes. Scale bar: 100 *μ*m. (b) The ALP activity as early marker of differentiation was associated with progressive mineralization of the extracellular matrix. Data shown are the mean ± SD of six replicas of three independent experiments (*n* = 18). Asterisks show significant differences with respect to negative control (hDPSC) (*p* < 0.05). (c) Absorbance measurement of alizarin red stain extracted at different stages of differentiation. Data shown are mean ± SD of six replicas of three independent experiments (*n* = 18).

ECM proteins, primarily COL-I, were synthesized beginning at seven days of differentiation as revealed by Masson's trichrome staining (Figure 7). As expected, the evaluation of ALP showed strong activity at early differentiation times (OLC-3 and -7) as a marker of early odontoblast differentiation but low expression (Figure 5) and activity in hDPSC, differentiated cells (OLC-21), and EXP-21 cells (Figure 8(b)). Calcium deposits and mineralization nodes progressively appeared during the differentiation process, as shown by alizarin red and von Kossa stainings, which revealed extensive calcified matrix in both the OLC and 14-day cultured EXP-21 cells (Figures 8(a) and 8(c)).

## 4. Discussion

This work showed that it is possible to differentiate dental pulp stem cells (or mesenchymal progenitor cells) into odontoblast-like cells. We also showed that the detachment, reseeding, and expansion of these differentiated cells did not affect the proliferative and differentiation characteristics of cultured OLC, which represents a valuable characteristic for their use in studies of regeneration or dental material biocompatibility. The most interesting advantage of the reported protocol is the possibility of obtaining a primary culture of relevant cells for research in dentistry, with a homogeneous and uniform phenotype that remains after detaching and reseeding, enabling researchers to perform reproducible experiments with many replicas and accurate cell number counting. The ability to freeze and thaw these cells for reuse on demand provides an additional benefit.

Many studies have reported that stem or progenitor cells of dental tissues are easy to obtain, can be expanded* in vitro*, and show remarkable plasticity; in addition, these cells differentiate into odontogenic cells [17]. The hDPSC isolated in this work showed classical mesenchymal stem cell markers similar to those obtained from human bone marrow, such as CD90, CD105, and CD73, as well as the absence of early hematopoietic markers. They also showed excellent proliferation and differentiation properties.

The proliferation and differentiation of hDPSC to odontoblast-like cells are known to depend on stimulation with specific differentiation media [18, 19]. Thus, we used TGF-*β*1 in addition to ascorbic acid, *β*-glycerophosphate, and dexamethasone. TGF-*β* is a multifunctional cytokine involved in cell growth, proliferation, and differentiation, as well as in extracellular matrix synthesis, indicating an important role for the signaling pathways involved in odontogenic differentiation; this includes the upregulation of the expression of noncollagenous proteins such as DSPP and DMP-1 [20], as supported by our results. The differential profiles of genes expression during odontoblast differentiation suggest that further lineage determination of mineralizing cells is the result of differences in signaling pathways that control gene expression in odontogenic differentiation.

We found that OLC had a population doubling time that was higher than those of hDPSC or EXP-21 cells, which is consistent with the notion that synchronization between the proliferation and differentiation of stem cells to specific lineages is a key aspect to maintain stem cell function. The conditions for inducing differentiation are not the same as those for inducing cell proliferation; therefore, during the start of differentiation, large gene expression changes occur so that the cells can acquire the specific functions of the differentiated cells [21]. There were no changes in the initial proliferation rates in either the hDPSC or EXP-21 cells, which both reached the plateau phase around the tenth day, possibly because the monolayer ceased proliferation due to high confluence and contact inhibition.

DSPP and DMP-1 have been suggested to be the main phenotypic markers of odontoblasts [7]. There was a correspondence between DSPP transcription and protein synthesis, as evaluated by qPCR and immunocytochemistry, respectively. The dentin sialoprotein (DSP) has been described to have specific temporal and spatial expression patterns within the developing and mineralizing dentin [1], although this role appears to be more important during* in vivo* studies than those of* in vitro* biomineralization and hydroxyapatite crystal deposition studies. For example, the insertion of the DSP gene in DSPP-null mice induced the recovery of dentin mineralization, which facilitated hydroxyapatite formation along the collagen fibrils. This process describes the predentin to dentin transition in the mineralization front [22].

Interestingly, we detected DSP located in the OLC nuclear compartment at early differentiation periods and in both fresh and cryopreserved EXP-21 cells, indicating a regulatory function during dentinogenesis. The cytoplasmic localization of DSP in differentiating OLC-14 and the extracellular staining in OLC-21 suggest active secretion during differentiation to promote the initial mineralization, connecting calcium ions to collagen fibers as is normal in dentin formation. The DSP protein and transcript expression of DSPP peaked after 14 days of differentiation, and this pattern was maintained in OLC-21 and in both fresh and thawed expanded cells (EXP-21). These results agree with those of Chen et al. (2008), who detected high DSP expression in mice MO6-G3 cells, indicating the differentiation of odontoblast cells; they also found staining in both the nuclei and cytoplasm [23]. However, the role of nuclear DSP remains to be elucidated.

Immunoperoxidase and immunofluorescence staining corroborated DMP-1 translocation from the nucleus to the cytoplasm and subsequent secretion to the ECM, a process clearly involved in the in vitro differentiation of odontoblasts. This protein plays an important role in odontogenesis, and its expression is required from early to late developmental stages. The role of DMP-1 signaling has been well studied; after endocytosis by preodontoblasts, DMP-1 induces calcium release from the endoplasmic reticulum, activating the p38 pathway and RUNX2 nuclear translocation, which are responsible for initiating the transcription of odontogenesis-related genes [24]. The nuclear/cytoplasmic localization of DSP and DMP-1 found in this work is compatible with the dual role regulator and nucleating proteins during ECM mineralization.

We described a substrate adherence pattern of OLC that may be important for optimizing and controlling the cellular microenvironment in surface engineering experiments [25]. Each cell type presented different affinities to ECM molecules depending on the expression of integrin *α* and *β*1 subunits [26]. EXP-21 cells had the best adhesion to collagen (I and IV), fibronectin, and fibrinogen but not to laminin, possibly due to the high expression of integrin *α*2, *α*6, and *β*1 induced by TGF-*β*1 treatment and the absence of *α*1, *α*3, and *α*7 necessary for laminin adherence, as reported by Warstat et al. (2010) [26].

ECM protein secretion and mineralization are the main characteristics of mature secretory odontoblasts. Our findings demonstrated that differentiation media upregulated the proteins necessary to establish a functional ECM. COL-I was highly upregulated at 7 days of differentiation, as observed in extracellular deposits. After 21 days of differentiation, all of the extracellular space was filled with fibers, with a structural band pattern present around each cell. Differentiation media induced increases in the size and number of mineralized nodes due to the upregulation of gene and ALP activity in the early stages of differentiation. It therefore appears evident that, in our cultures, there was an orchestration of multiple events leading the cells to synthetize suitable proteins and matrix elements to guarantee the controlled growth of hydroxyapatite crystals and the formation of a dentin matrix by OLC and both fresh and thawed EXP-21 cells.

EXP-21 cells after subcultivation did not show significant changes in the proliferation rate, the phenotype, or cell clustering. Large impacts on survival or phenotype are expected be associated with trypsin activity during detachment [27] due to the loss of the cell-ECM interaction and actin-myosin contraction. However, an enzyme plus mild EDTA treatment, such as that used in our study, could help maintain the viability and differentiation status of dissociated cells until medium with appropriate supplements restarts treatment, as has been reported for induced stem cells [28]. Interestingly, EXP-21 cells express 10-fold more OPN transcript than OLC-21. This ECM molecule is frequently induced by mechanical stress and is well recognized as a signal transduction activator when pulp repair is necessary [22, 29]. Therefore, EXP-21 cells can be suggested to have a differentiated phenotype like that present in active cells during the dentin matrix repair process.

In this culture system, maintaining the local microenvironment with the appropriate differentiation inducer signal also helps preserve the differentiated phenotype in subcultures. Therefore, the addition of differentiation medium containing TGF-*β*1 guarantees that the phenotype reached will be permanent in subcultures, preserving the odontoblast differentiation markers and the maintenance of ECM mineralization capacity observed in EXP-21 cells.

Based on the described findings, the reported* in vitro* model preserves most of the physiological, biochemical, and genetic characteristics of odontoblasts. Consequently, the expanded cells may be a useful tool for studying odontoblast differentiation and the underlying cell signaling. Further, we demonstrated that subcultivation, as well as cryopreservation and thawing of differentiated odontoblasts, is possible. Only minor changes in proliferation or differentiated phenotype were observed, suggesting the utility of subcultured cells in tissue engineering and biocompatibility studies on dental materials.

## Figures and Tables

**Table 1:** Primers used in this study.

Gene bank	Name	Forward
		Reverse
NM_001015051.3	*RUNX2*	5′-CATCTAATGACACCACCAGGC-3′
		5′-GCCTACAAAGGTGGGTTTGA-3′
NM_001040058.1	*OPN*	5′-TGAAACGAGTCAGCTGGATGACCA-3′
		5′-TGGCTGTGAAATTCATGGCTGTGG-3′
NM_001173467.1	*OSX*	5′-TGGGAAAAGGGAGGGTAATC-3′
		5′-CGGGACTCAACAACTCTGG-3′
NM_000478.4	*ALP*	5′-TCAGAAGCTCAACACCAACG-3′
		5′-GTCAGGGACCTGGGCATT-3′
NM_000088.3	*COLI*	5′-TGACCTCAAGATGTGCCACT-3′
		5′-ACCAGACATGCCTCTTGTCC-3′
NM_014208.3	*DSPP*	5′-GGCAGTGCATCAAAAGGAGC-3′
		5′-TGCTGTCACTGTCACTGCTG-3′
NM_004407.3	*DMP-1*	5-TGGAGTTGCTGTTTTCTGTAGAG-3
		5′-ATTGCCGACAGGATGCAGA-3′
NM_001101.3	*β-ACT*	5′-ATTGCCGACAGGATGCAGA-3′
		5′-GAGTACTTGCGCTCAGGAGGA-3′

RUNX2: runt-related transcription factor 2; OPN: osteopontin; OSX: osterix; ALP: alkaline phosphatase; COL-I: collagen type I; DSPP: dentin sialophosphoprotein; DMP-1: dentin matrix protein-1; *β*-ACT: *β*-actin.
